# Ion Channels as a Therapeutic Target: Drug Design and Pharmacological Investigation

**DOI:** 10.3390/ijms25010171

**Published:** 2023-12-22

**Authors:** Gabriella Guerrini, Maria Paola Giovannoni

**Affiliations:** NEUROFARBA, Pharmaceutical and Nutraceutical Section, University of Florence, Via Ugo Schiff 6, 50019 Sesto Fiorentino, Italy; mariapaola.giovannoni@unifi.it

This Special Issue intends to illustrate the novelties in the field of ion channels. The articles included in this Special Issue contribute to enriching all aspects of this topic, from recent research in the field of medicinal chemistry, pharmacology, and molecular modeling to the identification of new potential drugs, discussion on advances in our understanding of the involvement of ion channels in disease, and a better definition of ligand–protein interactions.

The research contribution by Ewa Szymanska et al. (contribution 1) reports a study on the synthesis and characterization of a novel series of compounds bearing the quinoxaline-2,3-diones core as ligands of GluK Kainate receptors (GluK1-5). The kainate receptors belonging to iGluR (ionotropic receptors for glutamate) family KARs are involved in various neurological and psychiatric disorders, but the role of the GluK3 subunit, compared to other KARs, is still poorly understood. A lack of pharmacological tools, the high homology between KAR and AMPA, and a lack of selective ligands has slowed down medicinal chemistry research, thus making the development of new compounds desirable. Since the 1980s–1990s, compounds containing the quinoxaline-dione scaffold (e.g., DNQX and NBQX) have been developed as AMPA/Kainate receptor antagonists, with the aim of obtaining compounds that are more potent, selective, and water-soluble. In this paper, the authors develop new compounds bearing at the 6-position a (hetero)aryl ethynyl moiety, as an elaboration of the quinoxaline-2,3-diones 6-substituted already found in their previously published work [1,2]. Among all of the products, compounds 27, 28, and 29 with pyrimidine, imidazodiazine, and pyrazolopyridine scaffolds, respectively, were highlighted due to their high-affinity on recombinant iGluK3 (range 0.13–0.28 μM); moreover, compound 28 stood out due to its selectivity in binding only GluK3 and its state of being completely inactive on the other examined receptors. Pharmacological functional tests (intracellular Ca^2+^ imaging assay) confirmed the antagonist profile in a dose-dependent manner (IC_50_ range 0.6–3.6 μM). Lastly, molecular modeling and molecular dynamics studies exploiting Cryo-EM receptors permitted the paper’s authors to identify the molecular determinants underlying the affinity of the chosen compounds. In particular, the 6-heteroaryl ethynyl substituent forms a stable p-p stacking with a loop of amino acids (Phe446, Thr741, Thr742, and Tyr745), but the determinant for GluK3 selectivity for compound 28 results in the non-conserved residue Asn722 being able to interact with the imidazodiazine ring with respect to the other receptors examined (i.e., GluK1) which possess Ser721.

Michela De Bellis et al. (contribution 2) report on extensive literature covering the structural basis for the Na_v_1.4 sodium channel block by small molecules. These channels regulate a wide number of physiological processes (for example, the maintenance of sodium homeostasis, action potential generation, muscle contraction, and neurotransmitter/hormone secretion); their dysfunction can cause many disorders in the central and peripheral nervous systems, the heart, and skeletal muscle, but it has been also evidenced that mutations in Na_v_ channels or their overexpression can also be implicated in tumor growth (including breast, colon, prostate, and non-small lung tumors). In addition, in various neuromuscular diseases (for example, congenital myasthenic syndrome, hypokalemic/hyperkalemia and normokalemic periodic paralysis, non-dystrophic myotonias, and enhanced or reduced excitability of muscles), the involvement of Na_v_1.4 channels has been evidenced. This evidence in its entirety amply justifies the conduction of research in this field, with the aim of obtaining selective channel blockers [3]. Of note, the authors’ research group has been studying the molecular requirements for Na_v_1.4 channel blockers through the synthesis of analogs of mexiletine and tocainide, which are drugs used in the treatment of myotonia and other muscular disorders. In this review, the authors report on the most interesting compounds (Figures 2 and 3 in contribution 2), with them identifying the very potent channel blocker To042 as the best analog of tocainide and compounds CI16 and VM11 as mexiletine analogs; the latter, in addition to their high channel blocker potency, showed an antioxidant effect, which could be synergic in the treatment of degenerative skeletal muscle disorders in which alteration in the Na_v_ channel occurs in combination with oxidative stress. 

An investigation into the role of voltage-operated calcium channels (VOCCs, CaV1.2) and intracellular calcium (iCa^2+^) in chondrogenic differentiation has been extensively investigated by Eiva Bernotiene et al. (contribution 3) in relation to osteoarthritis (OA) and its possible treatment. Mesenchymal stem cells (MSCs), mouse embryonic stem cells, and pluripotent stem cells (iPSCs) have been used for in vitro and in vivo assays on cartilage regeneration; they are able to generate chondrocytes, which are potentially useful in the treatment of OA. In addition to a number of cellular processes (i.e., cell growth and differentiation, secretion, and signaling pathways’ activation), intracellular calcium^2+^ seems to be involved in chondrogenesis and OA pathogenesis. In this paper, the authors investigate, using in vitro assays, the use of adult tissue-derived MSCs (such as menstrual blood-derived MSCs (MenSCs) and human bone marrow (BMMSCs)) in comparison to OA cartilage-derived chondrocytes; in particular, the researchers studied their potential chondrogenic differentiation, the role of iCa^2+^, and the effect of VOOC channel blockade on chondrocyte production [4,5]. The authors evidenced that the BMMSCs showed better chondrogenic differentiation potential with respect to MenSCs; however, a consistent cartilage regeneration effect in OA is still some distance away.

The study by Janos Almassy et al. (contribution 4) focuses on another type of calcium channel, the cardiac ryanodine receptor (RyR2), a ligand-gated Ca^2+^ release channel of the sarcoplasmic reticulum, which is involved in specific heart pathologies called cardiac ryanopathies. Among these pathologies, the authors discuss congestive heart failure (CHF), catecholaminergic polymorphic ventricular tachycardia (CPVT), calcium release deficiency syndrome (CRDS), and arrhythmogenic right ventricular dysplasia type 2 (ARVD2), all pathologies related to hyperactivity, mutation at different levels of the receptor, or redox modification of RyR2 [6]. High mortality in individuals suffering from these HFs is frequent, and therapies involving antiarrhythmic drugs afford sufferers little benefit in terms of survival. At present, there are no RyR2-specific inhibitors on the market, and current research strategies are focused on the chemical modification of drugs such as carvedilol (a β-blocker), flecainide (a sodium channel blocker), dantrolene (a hydantoin derivative used to treat malignant hyperthermia crisis), tetracaine, K201 (or JTV519, a 1,4-benzothiazepine acting as a suppressor of myocardial injury), therefore with different targets with respect to RyR2 [7]. The chemical modification of the aforementioned compounds has allowed for the production of new products such as VK-II-86, El20, and N-methylflecanide, which act at RyR2 channels and are promising agents for the treatment of CHF.

In a detailed review, the group of Yaroslav A. Andreev (contribution 5) depicts the involvement of transient receptor potential vanilloid subtype 3 (TRPV3) in calcium^2+^ homeostasis, and its overexpression is often associated with various pathological conditions. This receptor, discovered around 20 years ago, is involved in chemo- and thermosensation and hair growth in physiological conditions. A mutation in the *trpv3* gene causes genetic diseases (i.e., Olmsted syndrome), skin inflammation, and severe itching. In this paper, the authors report on the progress made in our understanding of TVRP3 and its interactions with ligands, with this knowledge being obtained through the use of new, high-resolution techniques (i.e., cryo-EM) [8]. The design and synthesis of TVPR3 antagonists are reported in numerous patents, in which there are also interesting results with regard to the administration routes utilized (transdermal or intradermal) and the various formulations. To date, examination of the few compounds that have entered clinical trials has been interrupted in the early phases of such trials, I or II, due to induced neuropathy.

The involvement of potassium channels in glucose metabolism related to the cancer glycome was analyzed in a study by Wawrzkiewicz-Jalowiecka A. et al. (contribution 6) The elevated intake of glucose by cancer cells and the high production of metabolites are two specific processes evidenced in cancer. The interconnections between potassium channels and cancer are complex due to the fact that different types of cancer modulate the activity of such potassium channels in different manners. The authors detail the mitochondrial functions and glycosylation in normal and cancer cells, with them finding modified expression (upregulation or downregulation) of microRNAs, which can indirectly mediate the expression of various potassium channels. In mitochondria, K^+^ channels demonstrate involvement in cytoprotective activity since the activation of these channels prevents mitochondrial super-oxide formation and reduces the risk of apoptosis. In contrast, ROS production increases in hypoxic conditions when there is lower activity of K+ channels. MicroRNA (short non-coding RNA molecules) is the linker between tumor cells, the microenvironment, and other cells [9], and it affects the expression of protein glycans and the potassium channels at the transcriptional level. Thus, the analysis of microRNA could be a valid cancer biomarker to further exploit.

Finally, the computational approach as a necessary tool for studying ion channels and their ligands is well illustrated by Kunze G. and Pliuschcheuskaya P. (contribution 7) At the same time, Fedida D. et al. (contribution 8) applied this approach (docking simulation, molecular dynamics, and leveraging steered molecular dynamics) to highlight the protein features of SARS-CoV-2 E and explain the binding between the inhibitors (hexamethylene amiloride derivative) and the protein itself. The first paper illustrates how, in the last 25 years, the number of ion channel structures resolved has significantly increased. In fact, after 1998, the receptor–ligand complex in its three-dimensional form was realized with X-ray and cryo-EM techniques. The authors illustrate the significant contribution of computational methods in drug design and the study of ion channels. The different computational approaches discussed in the research are the computer-aided drug design approach (CADD) used in ‘structure-based drug design methods’ and ‘ligand-based drug design methods’ and new approaches using molecular dynamic simulation, virtual docking techniques, and structure-based virtual screening to characterize ligand–ion channel interactions, predict ligand poses, binding energy, and pharmacological activity. Moreover, from the recent literature, the use of new protocols supported by artificial intelligence [10] for the virtual screening of ultra-large chemical libraries using the Deep Docking platform has been evolving among researchers.
